# Supplementary material for: Simultaneous proteome localization and turnover analysis reveals spatiotemporal features of protein homeostasis disruptions
Source: Nat Commun. 2024 Mar 11;15:2207. doi: 10.1038/s41467-024-46600-5 (PMC10928085; doi:10.1038/s41467-024-46600-5)
Supplement: Supplementary file 3 — Description of Additional Supplementary files [file 41467_2024_46600_MOESM3_ESM.docx]

**Description of Additional Supplementary Files**

**File Name:** Supplementary Data 1

**Description:** Abundance changes in ER stress vs. Normal AC16 cells

**File Name:** Supplementary Data 2

**Description:** Protein localization and assignment in Normal AC16 cell

**File Name:** Supplementary Data 3

**Description:** Turnover rate ratios in Thapsigargin vs. Normal AC16 cells

**File Name:** Supplementary Data 4

**Description:** Protein localization and assignment in Thapsigargin AC16

**File Name:** Supplementary Data 5

**Description:** Turnover rate ratios in Tunicamycin vs. Normal AC16 cells

**File Name:** Supplementary Data 6

**Description:** Protein localization and assignment in Tunicamycin AC16

**File Name:** Supplementary Data 7

**Description:** Protein localization and assignment in Normal iPSC-CM

**File Name:** Supplementary Data 8

**Description:** Turnover rate ratios in carfilzomib vs. Normal iPSC-CMs

**File Name:** Supplementary Data 9

**Description:** Protein localization and assignment in Carfilzomib iPSC-CMs

**File Name:** Supplementary Data 10

**Description:** List of canonical compartment markers in spatial experiments
